# Luteinizing Hormone and Testosterone Levels during Acute Phase of Severe Traumatic Brain Injury: Prognostic Implications for Adult Male Patients

**DOI:** 10.3389/fendo.2018.00029

**Published:** 2018-02-13

**Authors:** Alexandre Hohl, Fernando Areas Zanela, Gabriela Ghisi, Marcelo Fernando Ronsoni, Alexandre Paim Diaz, Marcelo Liborio Schwarzbold, Alcir Luiz Dafre, Benjamin Reddi, Kátia Lin, Felipe Dal Pizzol, Roger Walz

**Affiliations:** ^1^Centro de Neurociências Aplicadas (CeNAp), Hospital Universitário (HU), Universidade Federal de Santa Catarina (UFSC), Florianópolis, Brazil; ^2^Serviço de Endocrinologia e Metabologia, Departamento de Clínica Médica, Hospital Universitário (HU), Universidade Federal de Santa Catarina (UFSC), Florianópolis, Brazil; ^3^Serviço de Psiquiatria, Departamento de Clínica Médica, Hospital Universitário (HU), Universidade Federal de Santa Catarina (UFSC), Florianópolis, Brazil; ^4^Departamento de Bioquímica, Universidade Federal de Santa Catarina (UFSC), Florianópolis, Brazil; ^5^Intensive Care Unit, Royal Adelaide Hospital, Adelaide, SA, Australia; ^6^School of Medicine, University of Adelaide, Adelaide, SA, Australia; ^7^Serviço de Neurologia, Departamento de Clínica Médica, Hospital Universitário (HU), Universidade Federal de Santa Catarina (UFSC), Florianópolis, Brazil; ^8^Laboratório de Fisiopatologia Experimental, Universidade do Extremo Sul Catarinense (UNESC), Criciúma, Brazil

**Keywords:** traumatic brain injury, gonadotrophic axis, luteinizing hormone, testosterone, prognosis

## Abstract

Traumatic brain injury (TBI) is a worldwide core public health problem affecting mostly young male subjects. An alarming increase in incidence has turned TBI into a leading cause of morbidity and mortality in young adults as well as a tremendous resource burden on the health and welfare sector. Hormone dysfunction is highly prevalent during the acute phase of severe TBI. In particular, investigation of the luteinizing hormone (LH) and testosterone levels during the acute phase of severe TBI in male has identified a high incidence of low testosterone levels in male patients (36.5–100%) but the prognostic significance of which remains controversial. Two independent studies showed that normal or elevated levels of LH levels earlier during hospitalization are significantly associated with higher mortality/morbidity. The association between LH levels and prognosis was independent of other predictive variables such as neuroimaging, admission Glasgow coma scale, and pupillary reaction. The possible mechanisms underlying this association and further research directions in this field are discussed. Overall, current data suggest that LH levels during the acute phase of TBI might contribute to accurate prognostication and further prospective multicentric studies are required to develop more sophisticated predictive models incorporating biomarkers such as LH in the quest for accurate outcome prediction following TBI. Moreover, the potential therapeutic benefits of modulating LH during the acute phase of TBI warrant investigation.

## Traumatic Brain Injury (TBI) Epidemic

Traumatic brain injury is a worldwide significant public health problem and following an alarming increase in incidence has become the leading cause of morbidity and mortality of young male adults (1–6). In 2014–2015, the mean annual incidence of severe TBI hospitalizations, in the Brazilian city of Florianópolis (population 460,000), was 7.09/100,000 habitants with an associated hospital mortality of 3.54/100,000 habitants (Zanela et al., unpublished data). Among the survivors in our unit, 33.3% developed personality changes, 27.3% major depressive disorders, 15.1% anxiety disorders, and 9% alcohol dependence, significantly impacting quality of life (3) and work capacity (4). Long-term cognitive impairment is also highly prevalent, being clinically meaningful for more than 50% of our patients who survived a severe TBI (6, 7).

## Chronic Hypopituitarism and TBI

Described in 1918 (8), partial or complete hypopituitarism is a potential consequence of TBI (9, 10). There are several mechanisms by which TBI can cause hypothalamus-pituitary dysfunction, including hypoxic insult or direct mechanical injury to the hypothalamus, pituitary stalk or the gland itself, compression from hemorrhage, edema or increased intracranial pressure, and vascular injury to the hypothalamus or the pituitary gland (10–13).

Long-term morbidity in TBI survivors typically includes somatic, psychiatric, cognitive and neurological symptoms (3–5, 7, 14). These TBI-related sequelae can cover subtle signs of hypopituitarism, resulting in underestimation of posttraumatic hypopituitarism that also may aggravate the TBI-related morbidity itself (12). There are limited data available on TBI survivors regarding either the impact of hypopituitarism or the role of replacing hormones such as testosterone after TBI. Longer prospective multicentric studies are needed to monitor natural course of pituitary dysfunction in TBI and to follow long-term effects of hormonal replacement.

## LH as Biomarker of Mortality in Severe TBI

**Figure 1:**
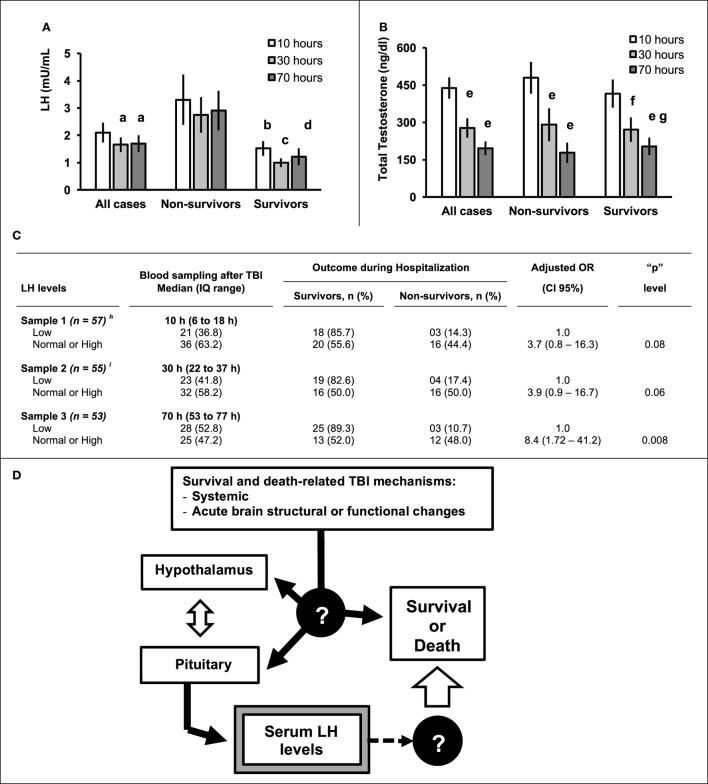
Serum level (mean ± SE) of luteinizing hormone (LH) **(A)** or total testosterone **(B)** determined in the Sample 1 (*n* = 57, median of 10 h after traumatic brain injury (TBI), IQ range = 6–18 h), Sample 2 (*n* = 55, median of 30 h after TBI, IQ range = 22–37), and Sample 3 (*n* = 53, median of 70 h after TBI, IQ range 53–77) according to hospital mortality. **(C)** Table showing the hospital mortality according to the LH levels adjusted for admission Glasgow coma scale and pupils’ examination. The normal or high LH levels in sample 1 (*p* = 0.08) and 2 (*p* = 0.06) showed a trend for independent association with mortality and a significant (*p* = 0.008) independent association in the Sample 3. **(D)** The survival and death-related TBI mechanisms may affects hypothalamus and hypophysis resulting in the LH levels changes (black arrows). It is unknown if the LH level itself affects the prognosis (dashed arrows) or is only an epiphenomenon without a cause-effect relationship with the patients’ prognosis. ^a^Significant difference (*p* = 0.04) between Sample 2 and Sample 1 and Sample 3 and Sample 1 by Paired-Samples “*T*” test; ^b^Significant difference between survivors and non-survivors for “*p*” < 0.02 (Sample 1) by Student “*t*” test; ^c^Significant difference between survivors and non-survivors for “*p*” = 0 0.002 (Sample 2) by Student “*t*” test; ^d^Significant difference between survivors and non-survivors for “*p*” = 0.01 (Sample 3) by Student “*t*” test; ^e^Significant difference from the Sample 1 for “*p*” < 0.0001 by Paired-Samples “*T*” test; ^f^Significant difference from the Sample 1 for “*p*” = 0.02 by Paired-Samples “*T*” test; ^g^Significant difference from the Sample 1 for “*p*” = 0.002 by Paired-Samples “*T*” test. ^h,I^Results previously published by Hohl et al. (10) and included as a part of his PhD Thesis in Medical Sciences (11).

The impact of hormone replacement for TBI-related hypopituitarism during the initial period after the injury depends on the type hormone deficiency. For example, unrecognized acute adrenocorticotropic hormone (ACTH) and cortisol deficiency or antidiuretic hormone deficiency (diabetes insipidus), can be life threatening (12). However, the prognostic implications for the low levels of luteinizing hormone (LH) and testosterone levels immediately following severe TBI remain incompletely understood. We reviewed the 10 articles available in PubMed (see Table 1) that evaluated LH and testosterone levels in adult male patients during hospitalization for severe TBI (9, 10, 14–21). In nine studies (9, 10, 13–21), hormone levels were determined in the first 10 days after TBI. In one study (14), levels were measured up to 20 days after the injury. Five studies included at least 30 male patients with severe TBI and analyzed LH and testosterone levels over 1 to 12 days after the injury (10, 14, 17, 18, 20). LH levels were low in 36.7–58.6% of patients and testosterone in 35.5–100% of patients. In our patients, both LH and testosterone levels decreased over the initial 3 days following the injury the (Figures 1A,B, respectively). In our patients, the Pearson’s correlation showed that in the first day the serum testosterone levels were significantly (*p* = 0.04) and positively correlated (*r* = 0.31) with serum testosterone (data not shown). There was no significant correlation between LH and testosterone levels on day 2 (*r* = 0.24, *p* = 0.09) or day 3 (*r* = 0.20, *p* = 0.18) post severe TBI (data not shown). The loss of this positive correlation suggests a time-dependent dissociation between LH and testosterone shortly after the injury.

**Table 1:** Previous studies showing gonadotrophic axis hormones profile during acute phase of severe TBI in male.

Reference	Time-course after TBI	Results in male	Clinical correlations and significance for mortality and morbidity
Agha et al. (14)	7 to 20 days (median 12)	Low testosterone levels in 79% of patients	Positive correlation between testosterone and TBI and GCS (*r* = 0.32, *p* = 0.048); Association between testosterone and prognosis were not analyzed
Cernak et al. (9)	Up to 7 days	Testosterone decrease in 2 days after TBI	Association between hormone levels and TBI severity and prognosis were not analyzed
Dalwadi et al. (15)	24 h	Low testosterone in 63.5% of patients	Correlation between testosterone and GCS did not reach significance No association between LH and testosterone and mortality Imbalances in the distribution of other predictors were not described Small sample size of severe TBI patients
Hohl et al. (10)	Up to 48 h	Low LH in 36.8% of patients in the 1st day and 41.8% in the 2nd day Low testosterone in 36.5% of patients in the 1st day and 73.1% in the 2nd day	Trend for independent association between normal or elevated LH mortality (*p* = 0.08) Distribution of other predictive variables and hormones were not controlled The possibility of type II error related to the small sample size should be considered
Kleindienst et al. (21)	Admission Day 1 and 7	In comparison to admission, the mean LH and testosterone levels decrease on day 3 and 7 after TBI	Association between injury severity and low testosterone and LH levels on day 3 Higher LH levels associated with number of lesions on the CT scan Association between testosterone and prognosis were not analyzed Small sample size of patients with severe TBI
Klose et al. (16)	Up to 12 days	Lower LH levels and testosterone	Correlation between low testosterone and TBI severity Wide range of time course between TBI and blood sampling for hormone analysis Association between hormones and prognosis were not analyzed Small sample size of patients with severe TBI
Hari Kumar et al. (17)	24 h	Low testosterone in 37.5% of patients	No association between LH or testosterone and GCS Association between hormones and prognosis were not analyzed Small sample size of patients with severe TBI
Olivecrona et al. (18)	Days 1 and 4	Low testosterone in 82.1 and 100% of patients at the 1st and 4th day after the TBI respectively Low LH in 55.2% and 58.6% of patients in the 1st and 4th days after the TBI respectively	Higher LH on day 1 (but not day 4) in patients with unfavorable outcome (morbidity and mortality) 3 months after TBI Regression models combining LH levels and ICPmax showed significant association with prognosis and ICPmax still was the main predicting factor (AUC for this model was not shown) Prognostic models including other predictors (GCS, pupils, sub-arachnoid hemorrhage) and LH levels were not analyzed by the authors
Tanriverdi et al. (19)	24 h	Significant lower testosterone levels, but not LH, in patients with severe TBI	Positive correlation between testosterone and GCS Association between testosterone and prognosis were not analyzed Small sample size of patients with severe TBI
Wagner et al. (21)	Day 1 to 9	Low LH in 50% of patients Low testosterone in 91% of patients LH and testosterone decline were seen in non-head injured extracranial trauma	LH or testosterone during acute phase of injury was not associated with the prognosis (GOS) 6 months after the TBI

*TBI, traumatic brain injury; GCS, Glasgow coma scale; GOS, Glasgow outcome scale; ISS, injury severity score; Marshal grade, Marshal Computed Tomography Classification; ICPmax, maximal intracranial pressure*.

Diagnostic and therapeutic decisions are based on a clinicians estimate of patient’s prognosis. Prognostic models, derived from statistical analysis of patient-related clinical, demographic, injury specific or biomarker variables, and clinical outcome, can increase the accuracy of clinical judgment regarding likely outcome following TBI (1, 5, 6, 22–24). We investigated whether serum levels of several hormones in the acute phase of severe TBI [Glasgow coma scale (GCS) ≤ 8], including free thyroxine (free T4), thyroid-stimulating hormone, LH, follicle-stimulating hormone, total and free testosterone, growth hormone, and insulin-like growth factor 1 were independently associated with hospital mortality in our patients (*n* = 60, all males) (10, 11). The protocol study was approved by the Human Research Ethics Committee of the Universidade Federal de Santa Catarina, UFSC (Process 163/2005 and 802.795). Written informed consent was obtained from the families. Gunshot injury victims and patients progressing to brain death within 24 h of admission were excluded. The primary outcome variable was hospital mortality. Additional variables analyzed were age, computed tomography (CT) findings, presence of associated trauma (thorax and/or abdomen), admission GCS score and pupil examination at admission. CT findings were classified in six categories according to Marshall classification and presence of subarachnoid hemorrhage. Hormone analyses undertaken at a median of 10 (IQR 6–18) h (*n* = 57) and 30 (IQR 22–37) h (*n* = 55) after TBI have been previously described (10). Additional analysis carried out in the same sample of patients 70 h after TBI are included in the present work (Sample 3, IQ range 53–77, *n* = 53). Of the investigated hormones, only LH showed a significant association with hospital mortality. As showed in Figure 1A, the mean level of serum LH was significantly lower in survivors than in non-survivors. After controlling for demographic, clinical, radiological, and neurosurgical variables by multiple binary regression, the association between normal or elevated LH levels observed at a median of 10 and 30 h after the injury and hospital mortality did not reach statistical significance when compared to patients with low LH levels (OR 3.7 *p* = 0.08 at 10 h and 3.9 *p* = 0.06 at 30 h Figure 1C). When LH levels were determined 70 h after the injury, normal or increased LH levels were significantly associated with hospital mortality when compared to lower LH levels (OR 8.4, CI 95% 1.72–42.2, *p* = 0.008). The observed association between normal or increased LH levels and hospital mortality was independent of the distribution of other investigated variables. Low GCS score and pupil abnormalities on admission also remained independently associated with mortality (not shown). Other studies evaluating gonadotrophic hormone levels in the acute phase of TBI are summarized in Table 1. Association between the outcome and hormone levels were not reported in five (9, 15, 17, 18, 20, 25). One study showed no association between the LH levels and prognosis, but a type II error related to the small sample size of cases with severe TBI needs to be considered (16). A Swedish study found a similar association between low LH levels in the acute phase of severe TBI and lower mortality and morbidity (*n* = 45; 30 males). They also evaluated a prediction model combining LH levels with intracranial pressure although other predictive variables like GCS and pupils’ abnormalities were not included in their model (Table 1) (19).

Another study including 88 patients with severe TBI investigated the association between LH levels determined in the acute phase and the Glasgow Outcome Scale (GOS) 6 months or more after the injury (21). The authors dichotomized the outcome according to the GOS score as favorable (>4) or unfavorable (≤4) where 5 = good recovery, 4 = mild disability, 3 = severe disability, 2 = vegetative state, and 1 = death. They found no association between LH level and 6 month GOS. In addition to the use of a biomarker to distinguish patients likely to have a good (GOS 5) vs. poor (GOS 2–4) outcome, a biomarker which can predict increased mortality would also be valuable. Accordingly, it would be interesting to analyze the predictive value of normal or higher LH levels for mortality in a patient cohort such as that studied by Wagner et al. (21). In our patients, LH levels determined earlier after the injury, alone or in combination with admission GCS or pupils’ examination, showed an elevated specificity (87–100%) but low sensitivity (<50%) to predict hospital mortality (data not shown).

Data regarding the relationship between the severity of TBI (based on the admission GCS) and testosterone levels are conflicted. Two studies including mild, moderate, and severe TBI showed a positive correlation between testosterone and GCS score (lower testosterone in lower GCS scores) (15, 17). However, this association was not confirmed by two other groups (16, 18). In studies including only severe TBI patients (10, 11, 19), or predominantly TBI patients (21), testosterone levels did not correlate with GCS. This may reflect the high percentage of abnormally low serum testosterone levels in patients included in the studies (21). Of note, severe TBI patients, testosterone levels neither correlate with Maximal Intracranial Pressure nor with the Minimal Cerebral Perfusion Pressure (19). Moreover, the association between testosterone levels and mortality or morbidity was significant in one study (19), not significant in four studies (10, 16, 21) and not analyzed in other six (9, 15, 17, 18, 20, 25).

## Does LH Level Affect Morbidity and Mortality in Severe TBI Patients?

Association studies can enhance diagnostic strategies, improve prognostic models, and highlight therapeutic targets of disease. If the observed findings in patients from Brazil (10, 11) and Sweden (19) are confirmed in other populations, LH levels can become a useful biomarker for mortality or morbidity in severe TBI patients. However, association studies do not prove a causative relationship between the predictive variable and the investigated end-point but can inform subsequent interventional, double blind, randomized, controlled, multicentre, studies designed to evaluate whether manipulating the gonadotrophic axis can improve outcomes from TBI.

The observed high/normal LH levels in patients at high risk of mortality could relate to a deleterious effect of LH, or a protective mechanism activated in patients with high risk for death. A third possibility is that changes in the LH levels during acute phase of severe TBI in males are merely a concomitant epiphenomenon with other physiological changes directly affecting the mortality in severe TBI.

The hypothalamus–pituitary axis can be directly injured through the mechanical impact or hemodynamic disturbance of a TBI. In addition, brain dysfunction related to secondary insults (6), including contributions from inflammatory, immunologic, and oxidative stress mechanisms (22, 23, 26, 27) can also affect the hypothalamus and pituitary by as yet unclear mechanisms affecting the severe TBI prognosis (Figure 1D).

As an example, we showed that elevated levels of the anti-inflammatory and neuroprotective interleukin IL-10 (>90 pg/mL) in the acute phase of severe TBI (10 and 30 h after the injury) was associated with 5 to 6 times higher risk of hospital mortality, independent of other variables including admission CT scan, GCS, and pupillary activity examination (23). One study previously provided a direct evidence in humans of a neuroanatomical pathway coupled with the peripheral immune system through IL-10 (28). The observed association between high IL-10 levels and higher mortality in our patients may occur, at least in part, because IL-10 can be a marker of “sympathetic” dysfunction in patients with severe TBI and worse prognosis. It was previously demonstrated that IL-10 levels increase when brainstem lesions result in sympathetic activation as catecholamines stimulate monocytes and trigger IL-10 release (28). A similar rationale may underlie the association of LH levels with mortality; sustained normal or elevated LH levels during the acute phase of severe TBI may relate to specific structural brain lesions or even systemic response also related to patients’ mortality.

It has been demonstrated that substance P (SP) induces LH release in animals and healthy men (29, 30). SP mediates the vascular permeability and edema formation that contributes to increased intra-cranial pressure after acute brain injury (31). Furthermore, serum levels of SP are associated with injury severity and mortality in patients with severe TBI (32). Taken together with the anatomical juxtaposition between the SP and gonadotrophin-releasing hormone (GnRH) systems in the human diencephalon, these results suggest a possible link between SP release and GnRH/LH release not only in physiologic (29, 30) but also pathological conditions like TBI (32). If LH itself affects the TBI prognosis or merely a marker of SP activity is an important point for further investigation.

Various animal models have been used for TBI research (33–35) and associations between TBI and hormone levels, including testosterone, have been replicated in rodents (36). However, drugs that have appeared to be neuroprotective in small animals have all failed to improve outcomes in human phase II or phase III clinical trials (31). Considering the complex mechanisms involved in the primary and secondary TBI-related hypothalamic and pituitary dysfunction, the use of TBI models including rodents (33, 34, 36) and large animals may be required to properly test the effects of LH and SP on TBI prognosis (35).

## Conclusion and Future Perspectives

The observation that hypogonadism is common in the acute phase of severe TBI in males across a range of different populations provides compelling evidence that this is true and potentially important phenomenon. Nevertheless, an association between testosterone levels and the male patients’ prognosis after TBI remains controversial. The association between normal or elevated LH levels in the acute phase of severe TBI and male mortality was replicated by two independent studies and the association with patients’ morbidity in one of them. The association between LH levels and prognosis in females, pediatric, and older patients remains unknown. If confirmed in other populations by prospective multicentre studies, the measurement of serum LH during the acute phase of injury has potential to become a useful prognostic marker of severe TBI. Prognostic models including LH levels during the acute TBI phase, in combination with other biomarkers, may also be useful to identify collinearity between LH levels and other neuro-humoral, immunologic, and endocrine biomarkers (27). Finally, the investigation of the LH as a therapeutic target in animal models of TBI is challenges for further translation research in TBI.

## Ethics Statement

The protocol study was approved by the Human Research Ethics Committee of the Universidade Federal de Santa Catarina, UFSC (Process 163/2005 and 802.795). Written informed consent was obtained from the families.

## Author Contributions

AH, FZ, GG, and MR, literature revision, data collection, manuscript writing; APD, MS, AD, KL, and FP, manuscript writing; RW, literature revision, manuscript writing, manuscript design, statistics analysis; BR, literature revision, manuscript writing, answer to reviewers.

## Conflict of Interest Statement

The authors declare that the research was conducted in the absence of any commercial or financial relationships that could be construed as a potential conflict of interest.
